# Adult mental health consequences of peer bullying and maltreatment in childhood: two cohorts in two countries

**DOI:** 10.1016/S2215-0366(15)00165-0

**Published:** 2015-06

**Authors:** Suzet Tanya Lereya, William E Copeland, E Jane Costello, Dieter Wolke

**Affiliations:** a Department of Psychology, University of Warwick, Coventry, UK; b Department of Psychiatry and Behavioural Sciences, Duke Medical Center, Sheffield, UK

## Abstract

**Background:**

The adult mental health consequences of childhood maltreatment are well documented. Maltreatment by peers (ie, bullying) has also been shown to have long-term adverse effects. We aimed to determine whether these effects are just due to being exposed to both maltreatment and bullying or whether bullying has a unique effect.

**Methods:**

We used data from the Avon Longitudinal Study of Parents and Children in the UK (ALSPAC) and the Great Smoky Mountains Study in the USA (GSMS) longitudinal studies. In ALSPAC, maltreatment was assessed as physical, emotional, or sexual abuse, or severe maladaptive parenting (or both) between ages 8 weeks and 8·6 years, as reported by the mother in questionnaires, and being bullied was assessed with child reports at 8, 10, and 13 years using the previously validated Bullying and Friendship Interview Schedule. In GSMS, both maltreatment and bullying were repeatedly assessed with annual parent and child interviews between ages 9 and 16 years. To identify the association between maltreatment, being bullied, and mental health problems, binary logistic regression analyses were run. The primary outcome variable was overall mental health problem (any anxiety, depression, or self-harm or suicidality).

**Findings:**

4026 children from the ALSPAC cohort and 1420 children from the GSMS cohort provided information about bullying victimisation, maltreatment, and overall mental health problems. The ALSPAC study started in 1991 and the GSMS cohort enrolled participants from 1993. Compared with children who were not maltreated or bullied, children who were only maltreated were at increased risk for depression in young adulthood in models adjusted for sex and family hardships according to the GSMS cohort (odds ratio [OR] 4·1, 95% CI 1·5–11·7). According to the ALSPAC cohort, those who were only being maltreated were not at increased risk for any mental health problem compared with children who were not maltreated or bullied. By contrast, those who were both maltreated and bullied were at increased risk for overall mental health problems, anxiety, and depression according to both cohorts and self-harm according to the ALSPAC cohort compared with neutral children. Children who were bullied by peers only were more likely than children who were maltreated only to have mental health problems in both cohorts (ALSPAC OR 1·6, 95% CI 1·1–2·2; p=0·005; GSMS 3·8, 1·8–7·9, p<0·0001), with differences in anxiety (GSMS OR 4·9; 95% CI 2·0–12·0), depression (ALSPAC 1·7, 1·1–2·7), and self-harm (ALSPAC 1·7, 1·1–2·6) between the two cohorts.

**Interpretation:**

Being bullied by peers in childhood had generally worse long-term adverse effects on young adults' mental health. These effects were not explained by poly-victimisation. The findings have important implications for public health planning and service development for dealing with peer bullying.

**Funding:**

Wellcome Trust, Medical Research Council, Economic and Social Research Council, National Institute of Mental Health, the National Institute on Drug Abuse, NARSAD (Early Career Award), and the William T Grant Foundation.

## Introduction

Child maltreatment is a global issue and has been a matter of intense public concern in high-income countries for more than a century.^1^ It has been defined as any physical or emotional ill-treatment, sexual abuse, neglect, or negligent treatment resulting in actual or potential harm to the child's health, survival, development, or dignity.^1^ Official estimates of confirmed cases range from 5·9% of children younger than 11 years in the UK^1^ to 12·5% children in the USA maltreated by 18 years of age.^2^ The risk for maltreatment is highest in the first few years of life.^2,3^ Exposure to maltreatment has been documented to have substantial physical health consequences^4^ and adversely affects mental health resulting in depression and anxiety disorders.^5^ It increases the risk for substance misuse^5^ and suicide attempts^6^ and has long-term effects on academic achievement and employment.^7^ Maltreatment alters biological stress systems, brain morphology, and networks that affect behaviour and control.^8^ Most governments in high-income countries have public policies to ensure that children are protected from violence and that all reasonable steps are taken to help them overcome adverse consequences.^9^

As children grow they spend more time with peers, and peer interactions take on increased importance.^10^ Peers are important for socialisation but can also be a substantial source of stress. Verbal and physical abuse and systematic social exclusion might be seen as peer maltreatment and are often described as bullying or peer victimisation. Bullying is characterised by repetitive aggressive behaviour engaged in by an individual or peer group with more power than the victim.^11^ It is a global issue; across 38 countries or regions, one in three children report being bullied.^12^ Like maltreatment, being bullied is reported to have adverse effects, including physical^13^ or mental health problems such as anxiety,^14,15^ depression,^16^ an increased risk of self-harm, and attempt or completion of suicide.^17,18^ Results from recent studies also show that being bullied can modify stress responses or lead to long-term increases in inflammatory processes.^19^ The effects on health and employment can last into early adulthood^20,21^ and even midlife.^20^

**Research in context**

**Evidence before this study**

We have run a systematic review in PsycINFO and Medline to identify potential literature published before Jan 5, 2015, using the search string “(bulli* or bully* or peer victimisation) and (abuse* or maltreat*) and (depress* or anx* or suic* or self-harm or mental health)”. We identified 172 peer reviewed articles in PsycINFO and 91 in Medline, none of which directly compared maltreatment and bullying.

**Added value of this study**

This is, to our knowledge, the first study to compare the long-term mental health outcomes of child maltreatment (by adults) with being bullied by peers. The results are consistent across the two cohorts (ALSPAC and GSMS) showing that children who were bullied by peers only were more likely to have overall mental health problems, anxiety, depression, and self-harm or suicidality than those who were neither bullied nor maltreated. Children who were both maltreated and bullied were also at increased risk for mental health problems, but the effects were not higher than those of being bullied alone. By contrast, our results did not show any increased risk of mental health problems for children that were maltreated (but not bullied) in the UK but showed an increased risk of depression according to the US cohort. Being bullied by peers had worse long-term adverse effects on young adults' mental health than being maltreated by adults.

**Implications of all the available evidence**

Both current results and previous literature show the negative effect of school bullying. The insufficiency of resources for bullying compared with those for family maltreatment requires attention. It is important for schools, health services, and other agencies to coordinate their responses to bullying, and research is needed to assess such interagency policies and processes. Future studies of maltreatment should take into account the effects of peer bullying.

In view of the similarity in long-term outcomes for bullying and maltreatment, it is reasonable to ask if the observed effects on bullied children are a result of experiencing both maltreatment and bullying, are attributable to previous maltreatment, or are independent of such maltreatment. Although previous studies have investigated the causes and outcomes of polyvictimisation,^22,23^ they did not directly compare the effects of maltreatment and peer bullying on mental health outcomes in young adults. The specific aim of the study was to compare the effects of maltreatment and peer bullying on mental health outcomes (ie, anxiety, depression, and self-harm or suicidality) in young adults in two large longitudinal samples.

## Methods

### Participants

We used data from the Avon Longitudinal Study of Parents and Children in the UK (ALSPAC) and the Great Smoky Mountains Study in the USA (GSMS) longitudinal studies. Table 1 shows similarities and differences between the ALSPAC and GSMS cohorts.

#### ALSPAC

ALSPAC is a birth cohort study set in western UK, examining the determinants of development, health, and disease during childhood and beyond.^26^ Briefly, women who were residents in Avon, UK, while pregnant and with an expected delivery date between April 1, 1991, and Dec 31, 1992, were approached to participate in the study. Of 14 775 livebirths, 14 701 (99%) were alive at 1 year of age. From the first trimester of pregnancy parents repeatedly completed postal questionnaires about themselves and the study child's health and development. Children were invited to attend annual assessment clinics, including face-to face interviews, and psychological and physical tests from age 7 years onward. The study website contains more details. We obtained ethics approval for the study from the ALSPAC Ethics and Law Committee and the Local Research Ethics Committees.

#### GSMS

The GSMS is a population-based sample of three cohorts of children, aged 9, 11, and 13 years at intake, recruited from 11 counties in western North Carolina, USA, in 1993, using a multi-stage household equal probability, accelerated cohort design.^27^ The first stage consisted of screening parents (n=3896) for child behaviour problems. All non-American Indian children scoring in the top 25% on a behavioural problems screener, plus a 1-in-10 random sample of the rest, were recruited for detailed interviews. All participants were given a weight inversely proportional to their probability of selection, so that the results are representative of the population from which the sample was drawn. This method meant that screen-high participants were weighted down and randomly selected participants were weighted up so that oversampling did not bias prevalence estimates. American Indian children were recruited with 100% probability. The study website contains more details.

### Predictor variables

#### ALSPAC

Maltreatment was assessed as physical, emotional, or sexual abuse, or severe maladaptive parenting (or both) between ages 8 weeks and 8·6 years as reported by the mother in questionnaires. Physical and sexual abuse was reported with two items answered by the mother (“he/she was sexually abused” and “he/she was physically hurt by someone”) at ages 1·5, 2·5, 3·5, 4·8, 6·8, and 8·6 years. The mother also reported two further questions (whether the “partner was emotionally cruel to child” and “partner was physically cruel to child”) at ages 8 weeks, 1·5 years, and 2·5 years. Abuse was coded as present if sexual, emotional, or physical abuse were reported at any time point.^17^ Severe harsh parenting (hitting, shouting, and hostility) was deemed present if children were exposed to maladaptive parenting at both preschool (from birth to up to 5 years of age) and school (age 5–8 years) years.^28^ Hitting was coded as present if it occurred daily or every week during preschool and often or sometimes during the school period. Shouting was coded as present if it occurred daily at preschool and often at school age. Lastly, hostility included “mum feels that whining makes her want to hit child”; “mum often irritated by child”; “mum has battle of wills with child”; and “child gets on mum's nerves”. Hostility was deemed as present if reported in three or all items. Maltreatment was a binary variable indicating presence versus absence of abuse or severe harsh parenting at any time from infancy to 8·6 years of age.

Being bullied was assessed with child reports at 8, 10, and 13 years using the previously validated Bullying and Friendship Interview Schedule.^16^ Frequency of being bullied was rated on a 4-point scale (0=never, 1=seldom, 2=frequently, 3=very frequently) across five types of overt (theft, threats or blackmail, physical violence, nasty names, nasty tricks), and four types of relational bullying (social exclusion, spreading lies or rumours, coercive behaviour, deliberately spoiling games). The range of scores for being bullied was 0–25 for 8 years (mean 3·1, SD 3·5) and 0–21 for 10 years (1·8, 2·6) and 13 years (1·8, 2·7). Children scoring 0 were classified as never bullied, those scoring 1–3 were classified as occasionally bullied, and those who scored 4 or more were classified as frequently bullied.^14^ Bullying by peers refers to the child reporting being frequently bullied (scoring 4 or more) at 8, 10, or 13 years of age.^14^

#### GSMS

Both maltreatment and bullying were repeatedly assessed with annual parent and child interviews between ages 9 and 16 years (up to eight assessments). Lifetime occurrence of physical and sexual abuse was assessed at every interview, whereas harsh parental discipline was assessed in the 3 months immediately preceding the interview. Maltreatment was present if child or parent reported that the child had been physically abused (participant victim of intentional physical violence by family member), sexually abused (participant involved in activities for purposes of perpetrator's sexual gratification), or the target of harsh parental discipline (defined as harsh, restrictive, or physical disciplinary style delivered coldly, or frequently in anger, unaccompanied by a generally nurturing atmosphere). The child and their parent reported on whether the child had been bullied or teased in the 3 months before the interview as part of the Child and Adolescent Psychiatric Assessment (CAPA).^24^ Being bullied was counted if reported by either the parent or the child at any assessment.

### Assessment of outcome variables

We derived ICD-10 diagnoses of anxiety and depression at age 18 years from a reliable and validated self-administered computerised version of the Clinical Interview Schedule (CIS-R).^25^ The CIS-R enables diagnoses according to the ICD-10 for common mental disorders. Computer algorithms are used to identify common mental disorders according to ICD-10 diagnostic criteria.^29^ Anxiety was a binary variable indicating presence versus absence of any generalised anxiety disorder, social phobia, specific phobia, panic disorder, or agoraphobia. Similarly, responses to questions were aggregated by the specified algorithm to derive a binary variable of ICD-10 diagnosis of depression.^30^

We assessed self-harm at 18 years from the CIS-R, with a binary variable coded from responses to the following two questions: “Have you ever hurt yourself on purpose in any way (eg, by taking an overdose of pills, or by cutting yourself)?” If yes, “How many times have you harmed yourself in the last year?” (not in the past year versus once, two-to-five times, six-to-ten times, or more than ten times).

We measured any DSM-IV anxiety disorder (generalised anxiety, agoraphobia, panic disorder, social phobia, obsessive-compulsive disorder, and post-traumatic stress disorder), depression, and suicidality (recurrent thoughts of wanting to die, recurrent suicidal ideation without a specific plan, suicidal plans or a suicide attempt) with the Young Adult Psychiatric Assessment (YAPA).^24^ Scoring programs, written in SAS 9.2, combined information about the date of onset, duration, and intensity of each symptom to create diagnoses according to the DSM-IV.^31^ 2-week test-retest reliability of the YAPA is similar to that of other highly structured interviews (κ for individual disorders ranged from 0·56 to 1·0). Validity is well established using multiple indices of construct validity.^24^ Overall mental health problems (any depression, anxiety, or suicidality) were recorded through ages 19–25 years.

### Potential confounders for ALSPAC and GSMS

Findings from a meta-analysis showed that family conflict, parent's level of stress, and parental mental health problems increased the risk of child abuse^32^ and being bullied.^28^ Hence, we controlled for sex of child, family hardships, and maternal mental health (appendix). For the ALSPAC cohort, the confounders were assessed during pregnancy. For the GSMS cohort, all confounders were assessed with annual parent and child interviews between ages 9 and 16 years. The appendix shows the association between all the variables included in the analyses.

### Statistical analysis

To identify the association between maltreatment, being bullied, and mental health problems, we ran binary logistic regression analyses and calculated odds ratios (ORs) with 95% CIs. The primary outcome variable was overall mental health problem (anxiety, depression, or self-harm or suicidality). Follow-up analyses were run for anxiety, depression, and self-harm. We analysed ALSPAC data using SPSS 20. For GSMS, we tested all models using SAS PROC GENMOD to run weighted regression models with robust variance (sandwich type) estimates derived from generalised estimating equations to adjust the standard errors for the stratified sampling design.

### Role of the funding source

The funders of this study had no role in study design, data collection, data analysis, data interpretation, or writing of the report. STL and DW had full access to the ALSPAC data and WEC had full access to the GSMS data. All authors made the decision to submit for publication.

## Results

In the ALSPAC cohort, 5217 participants attended the 18 year assessment and 4566 completed the mental health assessment. The current study included 4026 cohort participants (of whom 2239 were girls, 56%) who continued with the study at age 18 years and for whom data were available on early reports of maltreatment and bullying. Differences between current sample (n=4026) and the members from the ALSPAC cohort who were not included in the analyses can be found in the appendix.

In the GSMS cohort, of all 1777 participants recruited, 1420 (80%) agreed to participate. The weighted sample was 630 (49%) female. American Indian children were recruited with 100% probability; 350 (81%) of 431 recruited individuals agreed to participate. Of the 1420 participants recruited, 1273 (90%) were re-interviewed in young adulthood at ages 19, 21, or 24–26 years.

In the ALSPAC cohort, 775 (19%) of 4026 young adults in the sample had overall mental health problems (consisting of any depression, anxiety, or self-harm; table 2). 402 (10%) were classified as having anxiety, 316 (8%) as having depression, and 361 (9%) as having reported self-harm in the past year.

In the GSMS cohort (weighted percentages), 208 (18%) of 1273 young adults in the sample had overall mental health problems. 135 (12%) were classified as having anxiety, 87 (6%) as having depression, and 64 (7%) as having reported self-harm in the past year.

In the ALSPAC cohort, 341 (8%) of 4026 children were exposed to only maltreatment, 1197 (30%) were exposed to only bullying, and 283 (7%) were exposed to both maltreatment and bullying. Maltreated children were more likely to be bullied than children who were not exposed to maltreatment, (χ^2^ [1, n=4026]=23·5, p<0·0001). In the GSMS cohort, 207 (15%) of 1273 children were exposed to only maltreatment, 225 (16%) to only bullying, and 159 (10%) to both maltreatment and bullying. Similarly, maltreated children were more often bullied than those not maltreated (χ^2^ [1, n=1420]=67·2, p<0·0001).

Prospective associations between maltreatment by adults, being bullied, and mental health problems are presented in table 2 and adjusted results are presented in table 3. Compared with children who did not experience maltreatment or bullying, children who experienced maltreatment only were not more likely to have any mental health problems according to ALSPAC, and had more often depression according to the GSMS cohort. Children who were bullied by peers only were significantly more likely to have all mental health problems than were neutral children (those who did not experience maltreatment or bullying). Those who were both maltreated and bullied were more likely to have overall mental health problems, anxiety, and depression according to both cohorts and to have also reported self-harm or suicidality according to the ALSPAC cohort than were neutral children (table 2). After adjusting for potential confounders (table 3), being bullied only was a higher risk for overall mental health problem than was being maltreated only in both cohorts (OR 1·6 [95% CI 1·1–2·2] for ALSPAC; 3·8 [1·8–7·9] for GSMS). Specifically, children who were bullied were more likely to have anxiety (4·9 [2·0–12·0] for GSMS), depression (1·7 [1·1–2·7] for ALSPAC) and self-harm (1·7 [1·1–2·6] for ALSPAC) as adults than children who were maltreated by adults.

## Discussion

Our results consistently showed an increased risk of young adult mental health problems such as anxiety, depression, and self-harm or suicidality in children who were bullied by peers whether or not they had a history of maltreatment by adults. Maltreatment by itself did not increase the risk of any mental health problem according to the ALSPAC cohort and increased the risk of depression according to the GSMS cohort. When being bullied was directly compared with maltreatment in childhood, being bullied by peers had more adverse effects on early or young adult overall mental health. Maltreatment mainly had adverse effects on mental health problems when the children had also been bullied. Previous research has suggested that being bullied in childhood might be a marker for present and future risk of psychopathology and occurs above and beyond any pre-existing behaviour or emotional problem.^33^ A recent study^20^ showed that bullied children had similar risk of mental health problems as the risk for children who were placed in public or substitute care in childhood.^20^

Our findings showed that children who were exposed to bullying, whether previously maltreated or not, were more likely to have mental health problems in adulthood than those not exposed to either bullying or maltreatment. However, across both cohorts about 40% of children who were ever maltreated were also bullied. Experience of other forms of victimisation might create susceptibility for being bullied. According to the developmental victimology framework,^34^ many kinds of victimisation have common risk factors, such as family instability, insufficient supervision, and personal characteristics (ie, poor social interaction skills). Moreover, maltreatment by adults might interfere with children's emotional regulation,^35^ which might make them susceptible to being bullied. However, although children who were both maltreated and bullied displayed high levels of mental health problems, the effects were not higher than those of being bullied alone. This suggests that the effects of maltreatment on young adult mental health may be at least partly due to being bullied. Indeed, a recent study^36^ showed that the relationship between maltreatment and depression was mediated by overt and relational peer victimisation. Hence, bullying can be viewed as both a consequence of prior experiences, and also a cause or risk factor for subsequent mental health problems. Contrary to previous reports,^5,6^ our results showed that overall mental health problems are not due to maltreatment per se but present when children were also bullied. A reason for the lack of association may be that bullying takes place closer (up to age 13 years) to the onset of mental health problems assessed at 18 years compared to maltreatment (up to age 8 years) in the ALSPAC cohort. However, maltreatment and bullying were assessed at the same ages in the GSMS and maltreatment alone only increased the risk of depression. A further reason can be that the overall maltreatment variable might hide significant associations of specific abuse types with mental health. Indeed, when abuse types (physical, emotional, sexual, and severe harsh parenting) were analysed separately, sexual and emotional abuse were associated with mental health problems in adulthood (appendix). By contrast, physical abuse and harsh parenting had weak or no association with adult mental health.

It is important to note the strengths and methodological limitations of the study. The strengths of the study include the use of two prospective cohort studies based in the UK and USA with diverse populations in different social settings, allowing replication of findings; the use of multiple informants; the large sample sizes; and the availability of information regarding family hardships.

This study has several limitations. First, parents might under-report maltreatment.^37^ Therefore, the ALSPAC cohort was tracked over an 8 year period for investigation and placement of participants on the official child protection register.^3^ Parents who were investigated or registered for child abuse reported significantly more physical or emotional cruelty.^3^ However, only 3·9% of those reporting emotional cruelty and 6·6% of those reporting physical cruelty came to the notice of child protection agencies.^3^ It is generally recognised that reports of victimisation coming to the attention of professional agencies might even more severely underestimate the true rate of maltreatment.^38^ The results were similar in the GSMS cohort in which maltreatment was reported by both parent and child, and both cohorts had similar frequencies of maltreatment compared with previous studies of the same age groups.^1,2^ Second, the effects of maltreatment might be dependent on third variables such as exposures to toxins intra-uterine or frequent changes of caretakers and residual confounding cannot be excluded. Third, the effect of severity of maltreatment and age of onset were not investigated in this study. Future research should consider severity, chronicity, types, and onset of maltreatment. Fourth, in the ALSPAC cohort, not all children completed the mental health assessments at age 18 years. Those with higher family adversity and mothers with prenatal mental health problems were more likely to have dropped out (appendix). However, current study participants did not differ from those lost to follow-up on maltreatment or bullying experience. Empirical simulations show that even when dropout is correlated with predictor or confounder variables, the relation between predictors and outcome is unlikely to be substantially altered by selective dropout.^39^ Similarly, not all participants were interviewed at every assessment in the GSMS cohort, but the response rate remained high (>80%), and there was no evidence of selective dropout. Fifth, the GSMS is representative of children from the area sampled, but not of children in the US population. Finally, none of the cohorts took cyberbullying into account, but previous studies have shown a vast overlap between cyberbullying and traditional bullying.^40^

Our findings suggest that being bullied has similar and in some cases worse long-term adverse effects on young adults' mental health than being maltreated. The UN Convention on the Rights of the Child established that government is the main body responsible for prevention of and response to violence against children.^9^ All signatory nations are required to establish integrated child protection services,^9^ which allow early detection and enhance coordination between legal, medical, and service responses.^4^ Governmental efforts have focused almost exclusively on public policy to address family maltreatment; much less attention and resources has been paid to bullying. Since bullying is frequent and found in all social groups,^41^ and current evidence supports that bullied children have similar or worse long-term mental health outcomes than maltreatment,^36^ this imbalance requires attention. It is important for schools, health services, and other agencies to coordinate their responses to bullying, and research is needed to assess such interagency policies and processes. Future studies of maltreatment should take into account the effects of peer bullying.

## Figures and Tables

**Table 1:** Similarities and differences between the ALSPAC and GSMS longitudinal studies

	**ALSPAC**	**GSMS**
Type of study	Longitudinal birth cohort	Longitudinal, population-based community survey
Location	Avon, South West England, UK	North Carolina, USA
Population	14 701 children (alive at 1 year) and their families	1420 children and their parents
Available data collection points	Multiple datapoints since pregnancy until 18 years	Age 9 years through 16 years (early childhood), 19 years, 21 years, and 24–26 years (young adulthood)
Measurement of bullying	Child reports in interviews at ages 8, 10, and 13 years (Bullying and Friendship Interview Schedule)^16^	The child and their parent reported on whether the child had been bullied or teased or bullied others (part of Child and Adolescent Psychiatric Assessment)^24^
Measurement of maltreatment	Maternal reports in repeated questionnaires	Child and parent report in repeated interviews
Measurement of mental health problems	Standard clinical interviews for depression, anxiety, and self-harm (CIS-R)^25^	Interviews: Child and Adolescent Psychiatric Assessment (for 9–16 years) and Young Adult Psychiatric Assessment (for 24–26 years)^24^

ALSPAC=Avon Longitudinal Study of Parents and Children. GSMS=Great Smoky Mountains Study. CIS-R=Clinical Interview Schedule-Revised.

**Table 2:** Mental health outcomes of maltreatment and being bullied by peers

		**Overall mental health problem**			**Anxiety**			**Depression**			**Self-harm and suicidality**		
		n (%)*	OR (95% CI)	p value	n (%)*	OR (95% CI)	p value	n (%)*	OR (95% CI)	p value	n (%)*	OR (95% CI)	p value
**Maltreatment, being bullied, or both *vs* none (not maltreated nor being bullied)**													
ALSPAC (n=4026)		..	(n=4026)	..	..	(n=4026)	..	..	(n=4026)	..	..	(n=4026)	..
	None (n=2205)	339 (15%)	[reference]	..	175 (8%)	[reference]	..	116 (5%)	[reference]	..	156 (7%)	[reference]	..
	Maltreatment only (n=341)	59 (17%)	1·2 (0·9–1·6)	0·362	33 (10%)	1·2 (0·8–1·8)	0·276	25 (7%)	1·4 (0·9–2·2)	0·122	24 (7%)	1·0 (0·6–1·6)	0·980
	Being bullied only (n=1197)	296 (25%)	1·8 (1·5–2·2)	<0·0001	156 (13%)	1·7 (1·4–2·2)	<0·0001	135 (11%)	2·3 (1·8–3·0)	<0·0001	143 (12%)	1·8 (1·4–2·3)	<0·0001
	Both (n=283)	81 (29%)	2·2 (1·7–2·9)	<0·0001	38 (13%)	1·8 (1·2–2·6)	0·002	40 (14%)	3·0 (2·0–4·3)	<0·0001	38 (13%)	2·0 (1·4–3·0)	0·0002
GSMS (n=1273)		..	(n=1273)	..	..	(n=1273)	..	..	(n=1273)	..	..	(n=1273)	..
	None (n=682)	74 (11%)	[reference]	..	46 (6%)	[reference]	..	29 (2%)	[reference]	..	22 (5%)	[reference]	..
	Maltreatment only (n=207)	50 (17%)	1·7 (0·8–3·3)	0·16	24 (8%)	1·3 (0·6–3·1)	0·53	22 (9·5%)	5·6 (2·2–14·3)	<0·0001	15 (8·5)	1·9 (0·7–5·5)	0·23
	Being bullied only (n=225)	41 (36%)	4·7 (2·6–8·7)	<0·0001	34 (25·5%)	5·0 (2·4–10·3)	<0·0001	19 (11%)	6·9 (2·7–17·2)	<0·0001	14 (13%)	3·0 (1·2–8·0)	0·02
	Both (n=159)	43 (30%)	3·5 (1·7–7·1)	<0·0001	31 (26%)	5·1 (2·3–11·4)	<0·0001	17 (13·5%)	8·4 (3·1–22·7)	<0·0001	13 (10%)	2·2 (0·7–6·9)	0·19
**Maltreatment *vs* being bullied**													
ALSPAC (n=1538)		..	(n=1538)	..	..	(n=1538)	..	..	(n=1538)	..	..	(n=1538)	..
	Maltreatment only (n=341)	59 (17%)	[reference]	..	33 (10%)	[reference]	..	25 (7%)	[reference]	..	24 (7%)	[reference]	..
	Being bullied only (n=1197)	296 (25%)	1·6 (1·2–2·1)	0·004	156 (13%)	1·4 (0·9–2·1)	0·097	135 (11%)	1·6 (1·0–2·5)	0·037	143 (12%)	1·8 (1·1–2·8)	0·011
GSMS (N=432)		..	(n=432)	..	..	(n=432)	..	..	(n=432)	..	..	(n=432)	..
	Maltreatment only (n=207)	50 (17%)	[reference]	..	24 (8·3)	[reference]	..	22 (9·5)	[reference]	..	15 (8·5)	[reference]	..
	Being bullied only (n=225)	41 (36%)	2·9 (1·4–6·0)	0·006	34 (25·5)	3·8 (1·60–9·30)	0·003	19 (11·3)	1·2 (0·4–3·5)	0·71	14 (13·0)	1·6 (0·5–5·0)	0·42

OR=odds ratio. ALSPAC=Avon Longitudinal Study of Parents and Children. GSMS=Great Smoky Mountains Study. Being bullied only refers to being bullied by peers in at least one timepoint. Overall mental health problem refers to having anxiety, depression, or self-harm or suicidality. For GSMS: percentages are weighted; sample sizes are unweighted.

* Refers to the number of children who have the associated mental health problem.

**Table 3:** Mental health outcomes of maltreatment and being bullied by peers–adjusted analysis (all results are adjusted)

		**Overall mental health problem**			**Anxiety**			**Depression**			**Self-harm and suicidality**		
		n (%)*	OR (95% CI)	p value	n (%)*	OR (95% CI)	p value	n (%)*	OR (95% CI)	p value	n (%)*	OR (95% CI)	p value
**Maltreatment, being bullied, or both *vs* none (not maltreated nor being bullied)**													
ALSPAC (n=3904)		..	(n=3904)	..	..	(n=3904)	..	..	(n=3904)	..	..	(n=3904)	..
	None (n=2130)	330 (15%)	[reference]	..	171 (8%)	[reference]	..	113 (5%)	[reference]	..	150 (7%)	[reference]	..
	Maltreatment only (n=332)	56 (17%)	1·1 (0·8–1·5)	0·474	32 (10%)	1·2 (0·8–1·9)	0·304	23 (7%)	1·4 (0·9–2·2)	0·188	24 (7%)	1·0 (0·7–1·6)	0·857
	Being bullied only (n=1166)	285 (24%)	1·8 (1·5–2·2)	<0·0001	151 (13%)	1·7 (1·4–2·2)	<0·0001	129 (11%)	2·3 (1·8–3·0)	<0·0001	134 (11%)	1·7 (1·4–2·2)	<0·0001
	Both (n=276)	77 (28%)	2·1 (1·5–2·8)	<0·0001	38 (14%)	1·7 (1·2–2·6)	0·005	39 (14%)	2·9 (2·0–4·4)	<0·0001	35 (13%)	1·8 (1·2–2·8)	0·003
GSMS (N=1273)			(n=1273)	..	..	(n=1273)	..	..	(n=1273)	..	..	(n=1273)	..
	None (n=682)	74 (11%)	[reference]	..	46 (6%)	[reference]	..	29 (2%)	[reference]	..	22 (5%)	[reference]	..
	Maltreatment only (n=207)	50 (17%)	1·3 (0·7–2·6)	0·45	24 (8%)	1·1 (0·5–2·5)	0·89	22 (9·5%)	4·1 (1·5–11·7)	0·008	15 (8·5%)	1·7 (0·6–4·9)	0·32
	Being bullied only (n=225)	41 (36%)	4·7 (2·5–8·9)	<0·0001	34 (25·5%)	4·9 (2·3–10·4)	<0·0001	19 (11%)	5·8 (2·2–15·1)	<0·0001	14 (13%)	3·0 (1·2–7·7)	0·02
	Both (n=159)	43 (30%)	3·1 (1·4–6·8)	0·005	31 (26%)	4·5 (1·9–10·7)	<0·0001	17 (13·5%)	5·8 (2·0–17·2)	0·002	13 (10%)	2·2 (0·6–7·7)	0·24
**Maltreatment *vs* being bullied**													
ALSPAC		..	(n=1498)	..	..	(n=1498)	..	..	(n=1498)	..	..	(n=1498)	..
	Maltreatment only (n=332)	56 (17%)	[reference]	..	32 (10%)	[reference]	..	23 (7%)	[reference]	..	24 (7%)	[reference]	..
	Being bullied only (n=1166)	285 (24%)	1·6 (1·1–2·2)	0·005	151 (13%)	1·4 (0·9–2·1)	0·134	129 (11%)	1·7 (1·1–2·7)	0·030	134 (11%)	1·7 (1·1–2·6)	0·029
GSMS		..	(n=432)	..	..	(n=432)	..	..	(n=432)	..	..	(n=432)	..
	Maltreatment only (n=207)	50 (17%)	[reference]	..	24 (8%)	[reference]	..	22 (9·5%)	[reference]	..	15 (8·5%)	[reference]	..
	Being bullied only (n=225)	41 (36%)	3·8 (1·8–7·9)	<0·0001	34 (25·5%)	4·9 (2·0–12·0)	<0·0001	19 (11%)	1·3 (0·5–3·7)	0·60	14 (13%)	1·7 (0·6–5·3)	0·35

OR=odds ratio. ALSPAC=Avon Longitudinal Study of Parents and Children. GSMS=Great Smoky Mountains Study. Being bullied only refers to being bullied by peers in at least one timepoint. Overall mental health problem refers to having anxiety, depression, or self-harm/suicidality. For ALSPAC: adjusted for sex, family adversity during pregnancy and any prenatal maternal mental health problems (anxiety and/or depression). For GSMS: adjusted for sex, socioeconomic status, family instability and family dysfunction; and percentages are weighted; sample sizes are unweighted.

* Refers to the number of children who have the associated mental health problem.
