# Supplementary material for: Adult mental health consequences of peer bullying and maltreatment in childhood: two cohorts in two countries
Source: Lancet Psychiatry. 2015 Jun;2(6):524–31. doi: 10.1016/S2215-0366(15)00165-0 (PMC4580734; doi:10.1016/S2215-0366(15)00165-0)
Supplement: Supplementary appendix [file mmc1.pdf]

THE LANCET Psychiatry

Supplementary appendix

This appendix formed part of the original submission and has been peer reviewed. We post it as supplied by the authors.

Supplement to: Lereya ST, Copeland WE, Costello EJ, Wolke D. Adult mental health consequences of peer bullying and maltreatment in childhood: two cohorts in two countries. *Lancet Psychiatry* 2015; published online April 28. http://dx.doi.org/10.1016/S2215-0366(15)00165-0.

eTable 1: Selected Sample (n = 4026) vs Members ALSPAC who were not included (n = 10,625)

	Selected Sample n (%)	Full ALSPAC Cohort n (%)	Selected Sample vs. Full ALSPAC Cohort OR (95% CI), p
Gender			
Male	1787 (44.4)	5753 (54.0)	[reference]
Female	2239 (55.6)	4910 (46.0)	**1.5 (1.4 – 1.6), <.001**
Prenatal FAI, [a] mean (SD)	.8 (1.2)	1.1 (1.5)	**.8 (.8 – .8), <.001**
Prenatal maternal mental health problems			
No	2953 (75.6)	6241 (68.6)	[reference]
Yes	951 (24.4)	2853 (31.4)	**.7 (.6 – .8), <.001**
Maltreatment & Bullying			
None	2205 (54.8)	2301 (56.0)	[reference]
Maltreatment only	341 (8.5)	369 (9.0)	1.0 (.8 – 1.1), .653
Being bullied only	1197 (29.7)	1168 (28.4)	1.1 (1.0 – 1.2), .186
Both	283 (7.0)	270 (6.6)	1.1 (.9 – 1.3), .320

Abbreviations: OR, odds ratio; CI, confidence interval, [a] FAI, Family Adversity Index. Being bullied only refers to being bullied by peers in at least 1 time point.

eTable 2: Details of Potential Confounders

Confounders	Details
**<u>ALSPAC</u>**	
Family Adversity	Family adversity was measured with the Family Adversity Index[1] consisting of 17 items (e.g. financial difficulties, crime involvement, negative partner relationship, etc.) taken from questionnaires administered throughout pregnancy (8, 12, 18 and 32 weeks gestation). Any adversity that was present was rated as 1 and scores totaled (Grand Mean=0.8, SD=1.2).
Prenatal maternal mental health	Maternal mental health was measured with the Crown-Crisp Experiential Index (CCEI) for anxiety[2] and Edinburgh Postnatal Depression Scale (EPDS)[3] at 18 and 32 weeks gestation in pregnancy. Those who scored in the top 15% in CCEI[4,5] and/or those who scored 13 and above on the EPDS[6] at any time point were considered to suffer anxiety or depression problems (n = 951, 24.4%).
**<u>GSMS</u>**	
Low SES	Low SES was positive if the child's family met 2 or more of the following conditions: below the US federal poverty line based upon family size and income, parental high school education only, or low parental occupational prestige.[7] Overall, 582 (33.5) children had low SES.
Unstable family structure	Unstable family structure was positive if child's family met 2 or more of the following conditions: single parent structure, step-parent in household, divorce, parental separation, or change in parent structure. 453 (27.2) children had an unstable family structure.
Family dysfunction	Family dysfunction was positive if child's family met 5 or more of the following conditions: inadequate parental supervision of child's free time, over-involvement of the parent into the child's activities in an age-inappropriate manner, physical violence between parents, top 20% in terms of frequency of parental arguments, marital relationship characterized by absence of affection, apathy, or indifference, child is upset by or actively involved in arguments between parents, mother scores in elevated range on Mood and Feelings questionnaire, top 20% in terms of frequency of arguments between parent and child, and most parental activities are source of tension or worry for the child. 447 (28.1%) children had family dysfunction.

[1] Bowen E, Heron J, Waylen A, Wolke D. Domestic violence risk during and after pregnancy: findings from a British longitudinal study. *BJOG*. 2005;**112**:1083-89. [2] Crown S, Crisp AH. *Manual of the Crown-Crisp Experiential Index*. London: Hodder and Stoughton; 1979. [3] Cox JL, Holden JM, Sagovsky R. Detection of postnatal depression. Development of the 10-item Edinburgh Postnatal Depression Scale. *Br J Psychiatry*.1987;**150**:782-86. [4] Heron J, O'Connor TG, Evans J, Golding J, Glover V. The course of anxiety and depression through pregnancy and the postpartum in a community sample. *J Affect Disord*. 2004;**80**:65-73. [5] Ramchandani P, Stein A, O'Connor TG, Heron J, Murray L, Evans J. Depression in men in the postnatal period and later child psychopathology: A population cohort study. *J Am Acad Child Adolesc Psychiatry*. 2008;**47**:390-98. [6] Murray L, Carothers A. The validation of the Edinburgh Post-natal Depression Scale on a community sample. *Br J Psychiatry*. 1990;**157**:288-290.[7] Nakao K, Treas J. The 1989 Socioeconomic Index of Occupations: Construction from the 1989 Occupational Prestige Scores. *GSS Methodological Report No. 74*. Chicago, Illinois: National Opinion Research Center; 1992.

eTable 3. Association among Variables

Variables	1	2	3	4	5	6	7	8	9	10
**ALSPAC**										N/A
1. Maltreatment	---									N/A
2. Being bullied	.076***	---								N/A
3. Overall mental health problem	.035	.120***	---							N/A
4. Anxiety	.020	.079***	.682***	---						N/A
5. Depression	.041**	.113***	.598***	.343***	---					N/A
6. Self-harm/suicidality	.015	.087***	.643***	238***	.245***	---				N/A
7. Sex	-.070***	-.042**	.165***	121***	.114***	.114***	---			N/A
8. Prenatal family adversity [b]	t(4024) = -7.1***	t(4024)= -3.8***	t(4024) = -6.1***	t(4024) = -4.6***	t(4024) = -4.1***	t(4024) = -4.1***	t(4024) = -1.2	---		N/A
9. Prenatal maternal mental health [a]	.094***	.069***	.080***	.067***	.038*	.047**	.024	t(3902) = -13.1***	---	N/A
**GSMS**										
1. Maltreatment	---									
2. Being bullied	0.18***	---								
3. Overall mental health problem	0.07*	0.26***	---							
4. Anxiety	0.07*	0.27***	0.79***	---						
5. Depression	0.13***	0.17***	0.52***	0.29***	---					
6. Self-harm/suicidality	0.04	0.11***	0.59***	0.27***	0.29***	---				
7. Sex	-0.02	0.05	-0.13***	-0.11***	-0.11***	-0.05*	---			
8. Low SES	0.19***	0.11***	0.19***	0.13***	0.10***	0.11***	0.03	---		
9. Unstable family structure	0.22***	0.06*	0.04	0.05	0.05	-0.01	-0.03	0.23***	---	
10. Family dysfunction	0.23***	0.17***	0.09***	0.09***	0.18***	-0.01	-0.01	0.17***	0.30***	---

* Phi Correlation is significant at the 0.05 level (2-tailed); ** Phi Correlation is significant at the 0.01 level (2-tailed); *** Phi Correlation is significant at the 0.001 level (2-tailed). For ALSPAC n = 4026. [a] n = 3904, [b] compared by using two-sample t-tests. For GSMS n = 1273

eTable 4: Unadjusted and Adjusted Results - Mental Health Outcomes of Maltreatment and Being Bullied by Peers

	Overall Mental Health Problem		Anxiety		Depression		Self-Harm/Suicidality	
**Comparisons**	**OR (95% CI)**	**P-Value**	**OR (95% CI)**	**P-Value**	**OR (95% CI)**	**P-Value**	**OR (95% CI)**	**P-Value**
**Unadjusted Results**								
**ALSPAC**	(n = 4026)		(n = 4026)					
None	[reference]							
Maltreatment	1.2 (.9 – 1.6)	.362	1.2 (.8 – 1.8)	.276	1.4 (.9 – 2.2)	.122	1.0 (.6 – 1.6)	.980
Being bullied	1.8 (1.5 – 2.2)	<.001	1.7 (1.4 – 2.2)	<.001	2.3 (1.8 – 3.0)	<.001	1.8 (1.4 – 2.3)	<.001
Maltreatment x Being bullied	1.1 (.7 – 1.6)	.786	.8 (.5 – 1.4)	.511	.9 (.5 – 1.6)	.750	1.1 (.6 – 2.1)	.642
**GSMS**	(n = 1273)		(n = 1273)		(n = 1273)		(n = 1273)	
None	[reference]		[reference]		[reference]		[reference]	
Maltreatment	1.6 (.8 – 3.3)	0.18	1.3 (0.5 – 3.0)	0.60	5.6 (2.2 – 14.3)	<0.001	1.9 (0.7 – 5.5)	0.23
Being bullied	4.9 (2.6 – 8.9)	<0.001	5.1 (2.5 – 10.5)	<0.001	7.0 (2.8 – 17.4)	<0.001	3.0 (1.2 – 8.0)	0.02
Maltreatment x Being bullied	0.5 (.2 – 1.3)	0.15	0.9 (0.3 – 2.8)	0.78	0.2 (0.1– 0.9)	0.03	0.4 (0.1 – 1.9)	0.23
**Adjusted Results**								
**ALSPAC**	(n = 3904)		(n = 3904)		(n = 3904)		(n = 3904)	
None	[reference]		[reference]		[reference]		[reference]	
Maltreatment	1.1 (.8 – 1.5)	.474	1.2 (.8 – 1.9)	.304	1.4 (.9 – 2.2)	.188	1.0 (.7 – 1.6)	.857
Being bullied	1.8 (1.5 – 2.2)	<.001	1.7 (1.4 – 2.2)	<.001	2.3 (1.8 – 3.0)	<.001	1.7 (1.4 – 2.2)	<.001
Maltreatment x Being bullied	1.0 (.7 – 1.6)	.946	.8 (.5 – 1.4)	.475	.9 (.5 – 1.7)	.827	1.0 (.6 – 1.9)	.954
**GSMS**	(n = 1273)		(n = 1273)		(n = 1273)		(n = 1273)	
None	[reference]		[reference]		[reference]		[reference]	
Maltreatment	1.3 (.6 – 2.5)	0.52	1.0 (0.4 – 2.4)	0.99	4.1 (1.5 – 11.7)	0.007	1.7 (0.6 – 4.8)	0.33
Being bullied	4.8 (2.6 – 9.1)	<0.001	5.0 (2.4 – 10.5)	<0.001	5.9 (2.3 – 15.3)	<0.001	3.0 (1.2 – 7.7)	0.02
Maltreatment x Being bullied	0.5 (.2 – 1.5)	0.24	0.9 (0.3 – 3.2)	0.91	0.2 (0.1 – 1.0)	0.04	0.4 (0.1 – 2.2)	0.29

Abbreviations: OR, odds ratio; CI, confidence interval. Being bullied only refers to being bullied by peers in at least 1 time point. Overall mental health problem refers to having anxiety, depression and/or self-harm/suicidality. For ALSPAC: Adjusted results are controlled for sex, family adversity during pregnancy and any prenatal maternal mental health problems (anxiety and/or depression); For GSMS: Adjusted results are controlled for sex, SES, family instability and family dysfunction.

eTable 5: Descriptive Information about Maltreatment Groups

	Total	Overall Mental Health Problem	Anxiety	Depression	Self-Harm/Suicidality
	n (%)	n (%)	n (%)	n (%)	n (%)
**ALSPAC**					
Physical abuse	512/624 (82.0)	111/512 (21.7)	56/512 (10.9)	54/512 (10.5)	49/512 (9.6)
Sexual Abuse	15/624 (2.4)	9/15 (60.0)	4/15 (26.7)	5/15 (33.3)	4/15 (26.7)
Emotional Abuse	102/624 (16.3)	34/102 (33.3)	18/102 (17.6)	14/102 (13.7)	15/102 (14.7)
Harsh Parenting	51/624 (8.2)	12/51 (23.5)	7/51 (13.7)	9/51 (17.6)	4/51 (7.8)
**GSMS** [e]					
Physical abuse	193/366 (44.9)	47/193 (24.9)	31/193 (6.9)	22/193 (8.5)	16/193 (20.3)
Sexual Abuse	167/366 (43.7)	47/167 (27.8)	31/167 (16.9)	19/167 (14.2)	13/167 (10.4)
Harsh Parenting	122/366 (32.9)	22/122 (17.9)	16/122 (15.7)	9/122 (9.3)	7/122 (9.4)

For ALSPAC: there were 680 incidents of maltreatment and 55 individuals had more than one type of abuse; For GSMS: there were 482 incidents of maltreatment and 98 individuals had more than one type of abuse; Percentages are weighted; sample sizes are unweighted.

eTable 6: Difference between Maltreatment Groups on Young Adult Psychiatric Outcomes

	Overall Mental Health Problem		Anxiety		Depression		Self-Harm/Suicidality	
	OR (95% CI)	P	OR (95% CI)	p	OR (95% CI)	p	OR (95% CI)	P
**ALSPAC**								
**Physical Abuse**	(n = 4026)		(n = 4026)		(n = 4026)		(n = 4026)	
No	[reference]		[reference]		[reference]		[reference]	
Yes	1.2 (0.9 – 1.5)	.14	1.1 (0.8 – 1.5)	.44	**1.5 (1.1 – 2.0)**	**.02**	1.1 (0.8 – 1.5)	.61
**Sexual Abuse**	(n = 3999)		(n = 3999)		(n = 3999)		(n = 3999)	
No	[reference]		[reference]		[reference]		[reference]	
Yes	**6.4 (2.3 – 18.0)**	**<.001**	**3.3 (1.1 – 10.5)**	**.04**	**5.9 (2.0 – 17.5)**	**.001**	**3.7 (1.2 – 11.7)**	**.03**
**Emotional Abuse**	(n = 3968)		(n = 3968)		(n = 3968)		(n = 3968)	
No	[reference]		[reference]		[reference]		[reference]	
Yes	**2.1 (1.4 – 3.3)**	**<.001**	**2.0 (1.2 – 3.3)**	**.01**	**1.9 (1.1 – 3.4)**	**.03**	**1.8 (1.0 – 3.1)**	**.04**
**Harsh Parenting**	(n = 2713)		(n = 2713)		(n = 2713)		(n = 2713)	
No	[reference]		[reference]		[reference]		[reference]	
Yes	1.4 (0.7 – 2.6)	.37	1.5 (0.6 – 3.3)	.37	**2.7 (1.3 – 5.7)**	**.01**	0.9 (0.3 – 2.4)	.79
**GSMS**								
**Physical Abuse**	(n = 1273)		(n = 1273)		(n = 1273)		(n = 1273)	
No	[reference]		[reference]		[reference]		[reference]	
Yes	1.6 (0.9 – 3.0)	.14	**2.1 (1.0 – 4.2)**	**.04**	1.6 (0.7 – 3.9)	.25	1.0 (0.4 – 2.6)	.96
**Sexual Abuse**	(n = 1273)		(n = 1273)		(n = 1273)		(n = 1273)	
No	[reference]		[reference]		[reference]		[reference]	
Yes	**1.9 (1.0-3.6)**	**.04**	1.6 (0.8 – 3.3)	.23	**3.5 (1.4 – 8.4)**	**.006**	1.6 (0.6 – 4.6)	.35
**Harsh Parenting**	(n = 1273)		(n = 1273)		(n = 1273)		(n = 1273)	
No	[reference]		[reference]		[reference]		[reference]	
Yes	1.0 (0.4 – 2.2)	.99	1.4 (0.6 – 3.5)	.46	1.8 (0.6 – 5.7)	.31	1.4 (0.4 – 2.9)	.59

Abbreviations: OR, odds ratio; CI, confidence interval. Overall mental health problem refers to having anxiety, depression and/or self-harm/suicidality. For ALSPAC: there were 680 incidents of maltreatment and 55 individuals had more than one type of abuse; For GSMS: there were 482 incidents of maltreatment and 98 individuals had more than one type of abuse.
